# Is Desalination a Solution to Freshwater Scarcity in Developing Countries?

**DOI:** 10.3390/membranes12040381

**Published:** 2022-03-31

**Authors:** Nirajan Dhakal, Sergio G. Salinas-Rodriguez, Jamal Hamdani, Almotasembellah Abushaban, Hassan Sawalha, Jan C. Schippers, Maria D. Kennedy

**Affiliations:** 1 Water Supply, Sanitation and Environmental Engineering Department, IHE Delft Institute for Water Education, Westvest 7, 2611 AX Delft, The Netherlands; s.salinas@un-ihe.org (S.G.S.-R.); jancschippers@gmail.com (J.C.S.); m.kennedy@un-ihe.org (M.D.K.); 2 Wetsus, The European Centre of Excellence for Sustainable Water Technology, Oostergoweg 9, 8911 MA Leeuwarden, The Netherlands; 3 Faculty of Civil Engineering, Delft University of Technology, Stevinweg 1, 2628 CN Delft, The Netherlands; almotasembellah.abushaban@um6p.ma; 4 International Water Research Institute, Mohammed VI Polytechnic University, Lot 660, Hay Moulay Rachid, Ben Guerir 43150, Morocco; jamal.hamdani@um6p.ma; 5 Research Center of Excellence in Water, Energy and Environment Mechanical Engineering Department, Palestine Polytechnic University, Wadi Al-Haria, Hebron P.O. Box 198, Palestine; hsawalha@ppu.edu

**Keywords:** water scarcity, population growth, desalination, developing countries

## Abstract

Rapid population growth and urbanization are two main drivers for the over-abstraction of conventional freshwater resources in various parts of the world, which leads to the situation of water scarcity (per capita availability <1000 m^3^/year). Predictions based on the World Bank projected population data and the FAO AQUASTAT database for freshwater availability show that by 2050, 2 billion people living in 44 countries will likely suffer from water scarcity, of which 95% may live in developing countries. Among these, the countries that will likely be most strongly hit by water scarcity by 2050 are Uganda, Burundi, Nigeria, Somalia, Malawi, Eritrea, Ethiopia, Haiti, Tanzania, Niger, Zimbabwe, Afghanistan, Sudan, and Pakistan. Currently, these countries have not yet established desalination to meet their freshwater demand. However, the current global trend shows that membrane-based desalination technology is finding new outlets for supplying water to meet growing water demand in most of the water-scarce countries. These 14 water-scarce countries will demand an additional desalination capacity of 54 Mm^3^/day by 2050 in order to meet the standard of current municipal water demand and to compensate for the withdrawal of renewable resources. Case studies from India, China, and South Africa have highlighted that other countries may apply the strategy of using desalinated water for industrial users. Moreover, challenges to the widespread adoption of desalination exist such as expense, significant energy use, the need for specialized staff training, the large carbon footprint of facilities, environmental issues such as greenhouse gas emission (GHGs), chemical discharge, and operational problems such as membrane fouling.

## 1. Current Trends in a Global Desalination Industry

**Figure 1:**
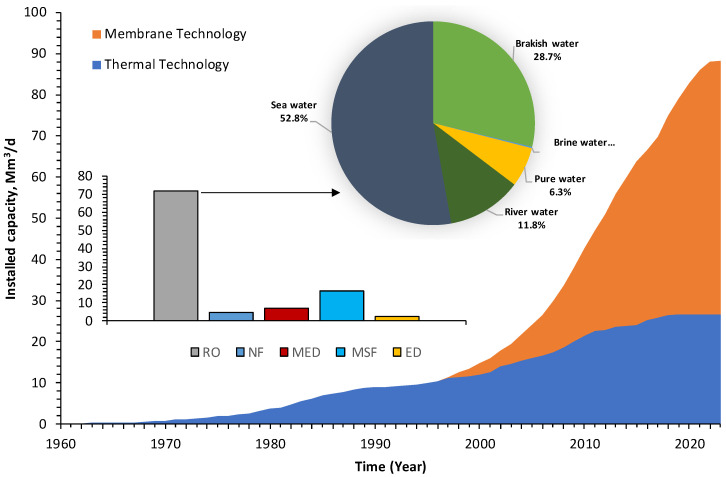
Global desalination capacity with regards to desalination technology and RO source water [3].

Large-scale seawater desalination began in the 1960s, using thermal distillation processes such as multi-stage flash (MSF) and multi-effect distillation (MED), which dominated the market until 2000 (Figure 1). The membrane-based technology reverse osmosis (RO) was introduced into the market in the 1970s, mainly to treat brackish water. Since the 1980s, advances in membrane technology and materials have made it possible to use RO technology for seawater applications [1]. As a result of this advancement, since 1999, membrane-based technologies, including RO, electrodialysis (ED), and nanofiltration (NF), have become the most dominant technologies for water desalination (Figure 1). Since that time, the average growth in desalination capacity throughout the world is about 7.5% per year, of which membrane desalination makes up about two-thirds of the total installed capacity [2]. The total desalination capacity (installed and projected, 2021) is about 115 Mm^3^/d, of which 77% (~88 Mm^3^/d) uses RO technology. In fact, the ratio is likely to change, since most new contracted desalination plants are founded on membrane-based technologies [3].

**Figure 2:**
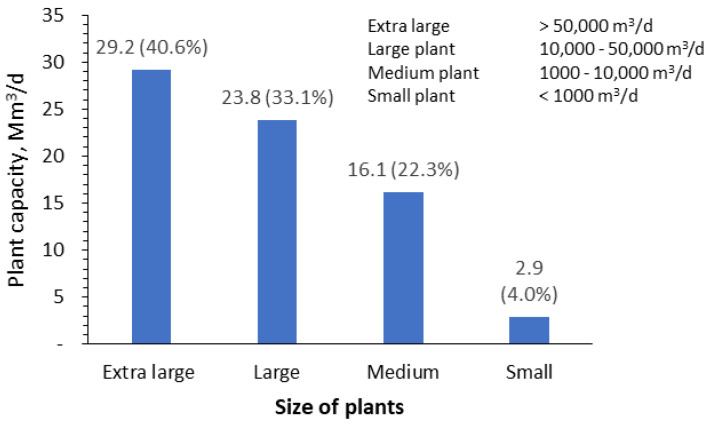
Classification of SWRO desalination plants based on their capacity [3].

**Figure 3:**
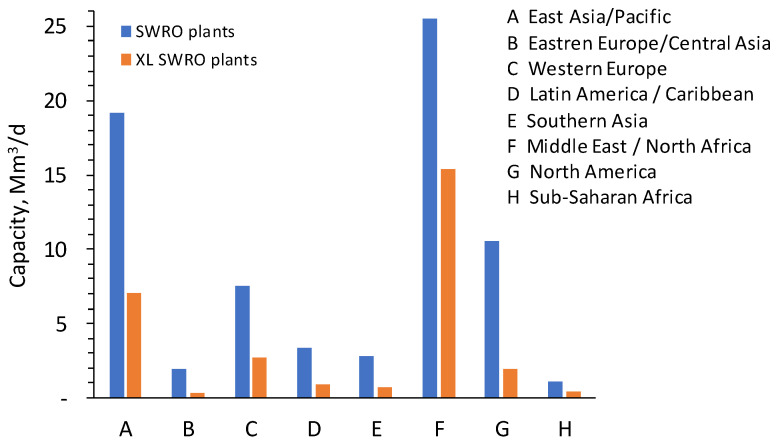
Total capacity of SWRO and share of extra-large plants in different regions of the world [3].

Based on the available data, RO is currently the most commonly applied technology, not only for seawater desalination, but also for drinking water and industrial water production. Almost half (53%) of the RO desalinated water is from seawater, and the rest is mainly from brackish, freshwater, and treated wastewater (Figure 1). Extra-large seawater RO (SWRO) plants (>50,000 m^3^/d) are already in service, comprising approximately 40% of the total installed capacity. The remaining plants are categorized as follows: 24% as large plants (10,000–50,000 m^3^/d), 15% as medium plants (1000–10,000 m^3^/d), and 3% as small plants (<1000 m^3^/d), as shown in Figure 2. Most of the extra-large plants are located in the Middle East and East Asia/Pacific, as illustrated in Figure 3.

**Figure 4:**
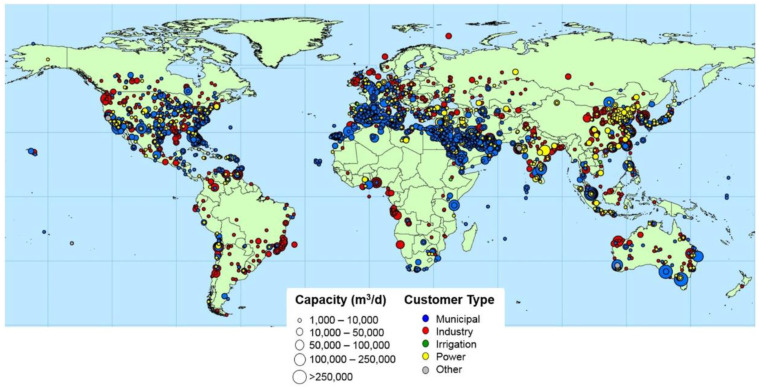
Global distribution of operational desalination facilities and capacities (>1000 m^3^/day) by sector user of produced water [5]. Reprinted with permission from ref. [5], 2019, Elsevier.

Figure 4 illustrates the currently installed desalination plants worldwide. It can be clearly seen that a high number of desalination plants have been installed in the Middle East, USA, Australia, China, Central Europe, the Mediterranean Region, and Japan. As indicated on the map, most of the desalination plants are located along the coastline, where desalination plants abstract raw water and where algal blooms frequently occur [4].

**Figure 5:**
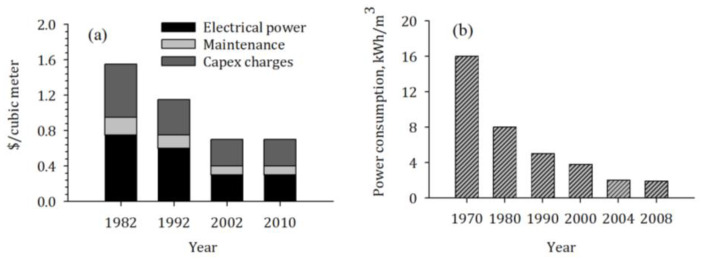
Trends of (**a**) cost in USD/m^3^ [9] and (**b**) power consumption in kWh/m^3^ [10] in seawater reverse osmosis plants.

The operational cost and energy consumption of membrane-based desalination have been reduced dramatically over time due to improvements in membrane technologies, energy recovery systems, and the use of renewable energy sources [6,7,8]. Figure 5a presents the costs required for electrical power, maintenance, and CapEx charges that decreased from USD 1.6/m^3^ in 1982 to USD 0.6/m^3^ in 2010. Moreover, Figure 5b shows that the energy consumption reduced from 16 kWh/m^3^ in 1970 to 1.9 kWh/m^3^ in 2008. 

## 2. Is There a Need for Desalination in Developing Countries?

**Figure 6:**
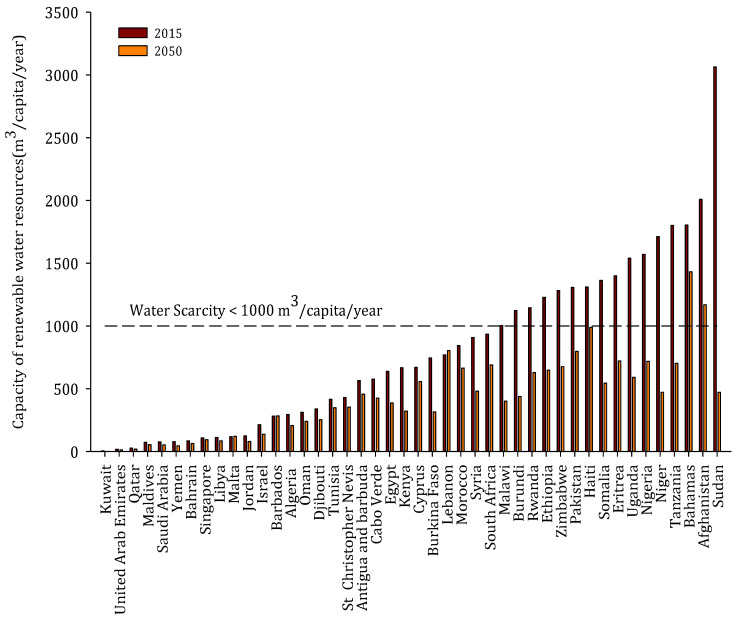
Countries expected to be water-scarce by 2050.

The concern over global water availability and its impacts has been expressed during the last decades using the alarming terms of “global water crisis” and “water scarcity” [11]. The economic and demographic growth are two main drivers for the over-abstraction of conventional freshwater resources in various parts of the world [12], leading to the situation of water scarcity. Water scarcity is usually considered as the situation when the total annual runoff available for human use is less than 1000 m^3^/capita/year [13]. As of 2015, 28 (mainly developing) countries are suffering from water scarcity. The situation of water scarcity is expected to worsen, given that by 2050, the population worldwide is anticipated to reach 9 billion. It has been estimated that by 2050, about 44 countries with a total population of approximately 2 billion people will likely suffer from water scarcity [2], of which 95% (1.9 billion) of those affected will live in developing countries. The majority of these countries are in Africa and Asia, namely, Malawi, Ethiopia, Sudan, Somalia, Nigeria, Uganda, Tanzania, Niger, Zimbabwe, Eritrea, Haiti, Burundi, Pakistan, and Afghanistan (Figure 6). The rapid increase in the population growth and the trend of rural-urban migration will intensify the issue of water shortage in these countries, mainly due to the withdrawal of fresh water to satisfy the demand for municipal and agricultural use [14]. The current available renewable resources in these countries are >1000 m^3^/cap/year, which will be drastically reduced to below 1000 m^3^/cap/year by 2050 due to the expected population growth (Figure 6). The estimation was based on the assumption that there will be no withdrawal of freshwater resources to fulfill the demand of the increased population. During the projection, the total available renewable water resources, which refer to the sum of actual groundwater and surface water in each country, was obtained from the FAO database [15], and the total populations (2020 and 2050) was acquired from the World Bank database [16]. 

The potential technical solutions to solve water scarcity are;
Saving waterIncreasing productivity in agriculture and industry 
Reducing leakages in public water supply 
Imposing progressive tariffs
Increasing rainwater harvesting Water transportTransporting from long distancesAquifer storageStoring river water during high flowWater reuseIncreasing reuse/recycling in industry
and domestic wastewater in agricultureDesalinationUsing brackish water, wastewater, seawater 

Among the different alternative solutions for solving the issues of water scarcity, desalination is only implemented as a last resort when conventional freshwater resources have been stretched to the limit. Desalination is considered as a drought-proof water source, since it does not depend on river flows, reservoir levels, or climate change. Desalination may be an option to alleviate scarcity in industry and for coastal cities. The report published by the United Nations showed that approximately 44% of the global population, and 8 out of the 10 largest metropolitan areas in the world, are located within a distance of 150 km from the coastline. The rate of population growth in the coastal regions is accelerating, and increasing tourism adds to the pressure on the environment (UN Atlas of the Ocean, 2017). Therefore, the possibility for widespread application of seawater desalination in the future is very likely [12]. Although the most well-known application of desalination (and related membrane technology) is to produce freshwater from seawater, it can also be used to treat slightly saline (brackish) water, low-grade surface and groundwater, and treated effluent resources [2]. The current global trend shows that desalination technology is finding new outlets as an alternative source for supplying water to meet growing water demands in most of the water-scarce countries [14]. However, there have been barriers to the widespread adoption of this technology, mainly due to its cost, energy requirements, a lack of expertise, and its carbon footprint.

## 3. Current and Future Status of Desalination Market in 13 Water Scare Countries 

The 13 countries that will be strongly hit by water scarcity by 2050 are Uganda, Burundi, Nigeria, Somalia, Malawi, Eritrea, Ethiopia, Haiti, Tanzania, Niger, Zimbabwe, Afghanistan, Sudan, and Pakistan (Figure 6). As shown in Figure 6, the current renewable water resources in these countries are greater than the threshold set for water scarcity (1000 m^3^/capita/year) and these are projected to be drastically reduced below the threshold by 2050. The current desalination status and the potential future market in these water-scarce countries have been studied. The current (2020) status of installed seawater and brackish water desalination plants (online, under construction, and presumed online) and its share for municipal and domestic purposes in each of the selected water-scarce countries is presented in Table 1.

As illustrated in Table 1, countries such as Burundi, Malawi, Uganda, and Zimbabwe have not yet installed any desalination plants, while other countries such as Afghanistan, Eritrea, Ethiopia, Niger, Nigeria, Pakistan, Somalia, Sudan, and Tanzania have already installed seawater and/or brackish water desalination plants. The user category in most of these countries that have already installed desalination plants is mainly for municipal water and industrial water use.

The current freshwater withdrawals in these 13 countries were studied based on the available data from FAO database. The general trend showed that most of the water-scarce countries withdraw freshwater mainly for agricultural activities, municipal use, and industrial use (Table 2). 

The highest use was in agriculture, which ranged from 0.02 to 9.13 m^3^/cap/day, with an average of 1.19 m^3^/cap/day. The average water withdrawal for municipal purposes was 0.140 m^3^/cap/day, which is eight times lower than for the agricultural sector. Table 2 shows that the current average per capita municipal and domestic water use is WW_AVG_ = 0.140 m^3^/cap/d. This was calculated from the municipal water withdrawals in each country and was distributed over the urban population in that country. The urban population is considered as a potential user of desalination in future. Based on this, the need for desalination capacity in these countries by 2050 was projected. The following assumptions were made during the projection.

1. No withdrawal of renewable water resources to meet the water demand by population growth.
2. The water demand required will only be supplied by desalination.
3. Only the populations of urban areas are assumed as the potential users of desalinated water.
4. The current average withdrawal for municipal purposes is assumed to be constant throughout the projection period.

The potential desalination (seawater and brackish water) growth in each of the selected water-scarce countries was calculated using the difference between the desalination capacity (Q2050) and the currently installed desalination capacity (Q2020) using Equations (1) and (2) [14]

∆Q_2050_ = Q_2050_ − Q_2020_ = (N_2050_ U.WW_2050_ ) − (Q_SW_ Y_SW_ + Q_BW_ Y_BW_) (1)

Q_2020_ = (Q_SW_ Y_SW_ + Q_BW_ Y_BW_)/(N_2020_ U) (2)

where,

N_2050_ = projected population by 2050 (World Bank)

N_2020_ = population in each country in 2020

U = population share that lives in urban centers 

WW_2050_ = per capita municipal and domestic water use by 2050 in m^3^/cap/d

Q_SW_ and Q_BW_ = currently installed sea and brackish water desalination capacity, m^3^/d

Y_SW_ and Y_BW_ = share of capacity used for municipal water production.

The projected growth in desalination capacity in the selected water-scarce countries for the coming 40 years is summarized in Table 3. The current total population in these 13 countries is 819 million, which is expected to increase to 1252 million by 2050, of which approximately 10–50% will live in urban cities. By 2050, a desalination capacity of 57.1 Mm^3^/d will be required to maintain the current per capita water demand (0.140 m^3^/cap/d) and to compensate for the freshwater withdrawals. This indicates the growth of the desalination market to be 54 Mm^3^/d, which is approximately a 1464% increase as compared to the current installed capacity (2.4 Mm^3^/d) in these 13 countries. However, there are challenges for the implementation of the desalination technologies in these countries that still need to be overcome. 

## 4. Case Study

**Figure 7:**
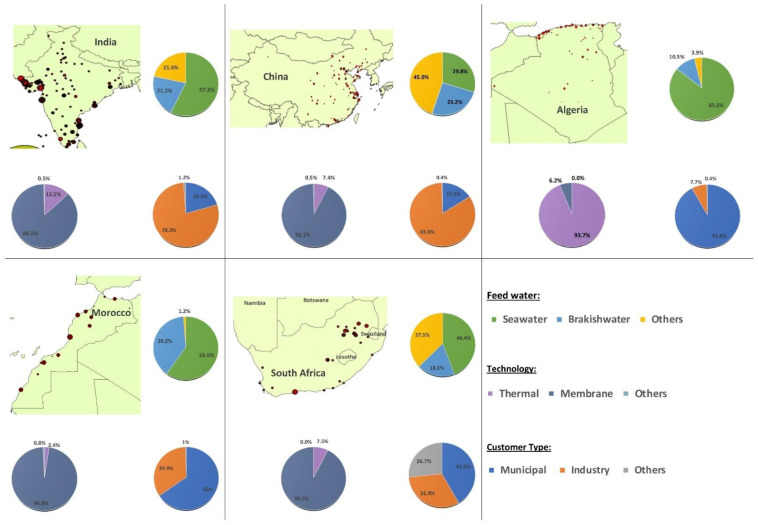
Country comparison of desalination use in India, Algeria, China, and South Africa [3].

A closer analysis of countries where a significant installed desalination capacity already exists, such as India (2.87 Mm^3^/d), China (9.72 Mm^3^/d), Algeria (2.76 Mm^3^/d), Morocco (0.27 Mm^3^/d), and South Africa (0.45 Mm^3^/d), was performed (Figure 7). The analysis was carried out based on the use of raw water sources (seawater, brackish water, or others), user categories (municipal, industrial, or others), and the type of technology used, i.e., membrane (RO, ED, and NF), thermal (MSF, MED), or others.

Among the four selected countries, India and China currently have total available renewable water resources which are higher than the threshold level of water scarcity (1000 m^3^/cap/year); these are distributed to a total population of approximately 1350 million people. However, the current trend of rural-urban migration and the unequal distribution of the renewable water resources have led to regional water crises in both of these two countries. This problem was the driving force for these countries to treat unwanted water such as seawater, brackish water, and wastewater using desalination technology. The currently installed desalination capacity in India is 2.87 million m^3^/d, and in China, it is 9.72 million m^3^/d. In both countries, membrane-based desalination leads thermal-based desalination, and 75% of the desalinated water is used for industrial purposes.

The other selected countries, such as South Africa, Morocco, and Algeria, currently have total available renewable water resources below 1000 m^3^/cap/year, distributed to a total population of approximately 80 million. Currently, Algeria and Morocco suffer from a severe water scarcity, which has forced them to abstract all available renewable water resources. However, South Africa, on the other hand, has tried to maintain its available freshwater resources, even though it has been categorized as a water scarce country [15]. All three countries have started to treat their water using desalination technology in order to meet the growing water demand. As of 2020, the installed total desalination capacity is 2.76 million m^3^/d in Algeria, 0.27 million m^3^/d in Morocco, and 0.45 million m^3^/d in South Africa. In Algeria, more than 90% of the desalinated water is used for municipal purposes, while in Morocco and South Africa, about 30% of the desalinated water is used for industry.

Likewise, Palestine is another interesting case in this regard. With total available renewable water resources of around 185 m^3^/cap/year in 2015, which is projected to decrease to 83 m^3^/cap/year in 2050 [17], the country faces a real water crisis. The water scarcity in Palestine is a result of both natural and man-made constraints related the specific Palestinian political situation. In the Gaza strip, one of the most densely populated areas in the world, almost 97% of the available renewable water resources are considered unfit for human consumption [18]. This is due to the over-extraction of water from the available aquifer, seawater intrusion, and infiltration of sewage and chemicals. The country identified desalination as a major strategic option for providing 2 million Palestinians in the Gaza strip with potable drinking water. About 70% of the drinking water needs of the Gaza strip is projected to be covered through desalination. To achieve this, the Palestinian water authority created a plan to increase the total desalination capacity from approximately 0.011 Mm^3^/d in 2015 to 0.35 Mm^3^/d by year 2032 [19].

Overall, the strategy adopted by India and China, i.e., to supply desalinated water for industrial use, could be applied to other countries to solve the issue of water scarcity by 2050.

## 5. What Are the Challenges?

**Figure 8:**
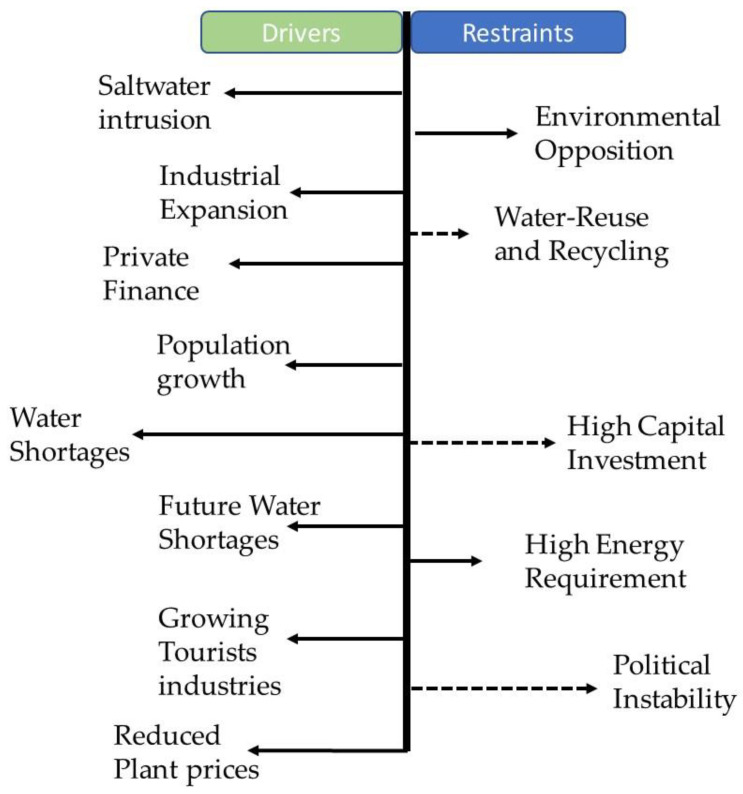
Key desalination market forces [23].

Desalination is “often chemically, energetically and operationally intensive, focused on large systems, and thus requires a considerable infusion of capital, engineering expertise, and infrastructure” [20]. The main “Achilles heel” for the efficient operation of a membrane-based desalination systems is membrane fouling [21]. The problems associated with membrane fouling are decreased membrane permeability, increased operating pressure, increased frequency of chemical cleaning, and membrane deterioration [22]. Despite all these facts, desalination is gaining a market to meet the demand of freshwater shortage worldwide. However, several factors that drive the feasibility of desalination plants need to be considered. Figure 8 indicates the drivers and restraints of the desalination market. As illustrated, the main drivers for desalination markets are saltwater intrusion, the willingness of private investors to invest, water shortages, reduced plant prices, etc. On the other hand, the environmental impact, high capital cost, and political instability are main restraints on the desalination market. As illustrated in the Figure 8, the significance of each factor (for both drivers and restraints) is indicated by the length of the arrow. The longer the arrow in the figure, the higher the importance of the factor. Contrastingly, the dotted arrows highlight the forces whose importance is gradually decreasing, for instance, factors such as high capital investment are decreasing, as the operational cost and energy consumption of membrane-based desalination have been reduced dramatically over time due to the improvements in membrane technologies, energy recovery systems, and the use of renewable energy sources [6,7,8,23]. This drastic decline in operational costs encourages investors to invest more in desalination projects. In summary, the most important driver for desalination projects is mainly water shortages due to the depletion of freshwater because of high water demand (population growth, industrial expansion) and increasing saltwater intrusion. 

It is therefore recommended to perform the PESTLE Risk Analysis, which includes analysis of various factors (political, economic, social, technological, legal, and environmental) that have a direct or long-lasting impact on processes and technologies. This analysis identifies opportunities and external risks that must be considered and not ignored [24,25]. 

### 5.1. Economic Issue 

Desalination is still the most energy-intensive technology for producing drinking water, and it is usually only implemented as a last resort when conventional freshwater resources have been stretched to the limit. The global concerns over climate change, water scarcity, rapid urbanization, and industrialization are some factors that directed many scientists and engineers to think of desalination to meet the demand of freshwater supply worldwide [26]. However, the costs required to produce and distribute freshwater from seawater through desalination is still a matter of debate when compared with the costs associated with conventional water supply systems (coagulation, flocculation, sedimentation, and filtration scheme). As illustrated in Table 4, the cost of desalinated water was approximately two times higher compared to the cost of the conventional water supply. Likewise, the cost related to energy consumption was also approximately 5–25 times higher for desalinated water compared to conventionally treated water (Voutchkov, 2011, 2014; Plappally, 2012) and cited by [26].

In most of the urban cities, the available freshwater water resources have reached the capacity limit mainly because of population growth and urbanization. This circumstance has forced many cities to treat their brackish water, seawater, and wastewater via desalination or to transport their freshwater from long distances. The choice depends upon the cost requirements or the decisions of the governments of the respective countries. Gude (2016) compared the relative average cost for providing drinking water in various countries using conventional treatment [26]. For instance, people in Beijing, China, pay the average cost of about USD 1.13/m^3^ for desalinated municipal water. The desalinated seawater in Beijing is collected from a distance of 135 km, with an elevation difference of 100 m from the source to the distribution site. Likewise, in Delhi, India, the cost for the desalinated water is about USD 1.9/m^3^. In this city, the desalinated water is transported from a distance of 1050 km, with an elevation difference of 500 m. On the other hand, the costs paid for municipal water by most European citizens are much higher compared to the costs paid by citizens from developing countries. The difference in the cost for water could be due either to government policy or due to strict environmental and economic standards of these countries. For instance, the lower water prices in India and China compared to European countries could be related to the fact that, in these countries, the water prices are highly subsidized by the government [26].

The detailed challenges and opportunities associated with the economics of desalination are provided by [7,27]).

### 5.2. Environmental Issues

The desalination plants have a significant environmental impact. Despite many efforts, there are still some environmental concerns [28] such as:

- disposal of material use
- land use
- energy use to desalinate water and greenhouse gas (GHGs) emissions
- brine discharge
- high volume of chemical use
- loss of aquatic organisms from marine pollution and open seawater intake

The use of fossil fuels to desalinate water emits greenhouse gases, including carbon monoxide (CO), nitric oxide (NO), nitrogen dioxide (NO_2_), and sulfur dioxide (SO_2_). The recent technological advances have helped to decrease the emission of GHGs; this decrease depends on whether oil is used instead of natural gas [29]. Likewise, the use of a high volume of chemicals during the pre- and post-treatment of seawater is another environmental concern. The main concern is the discharge of chemicals into the natural water, which affects the ecological imbalance [28]. Furthermore, the design of open seawater intake has a potential role in the loss of aquatic organisms, as these organism sometimes collide with the intake screen or can be drawn into the plant [29]. Some of the possibilities for sustainable solutions to prevent/minimize the issue listed above are [28]:

- implement low or no chemical technologies
- treat the chemicals before discharging into the natural water bodies
- disperse the concentrate through a multiport diffuser in a suitable marine site
- use subsurface or submerged intakes with low intake velocities
- reuse of material
- recover salts from the brine (resource recovery)
- use renewable energy sources to partially/completely fulfil the energy requirements
- use energy recovery devices to recover hydraulic energy

**Figure 9:**
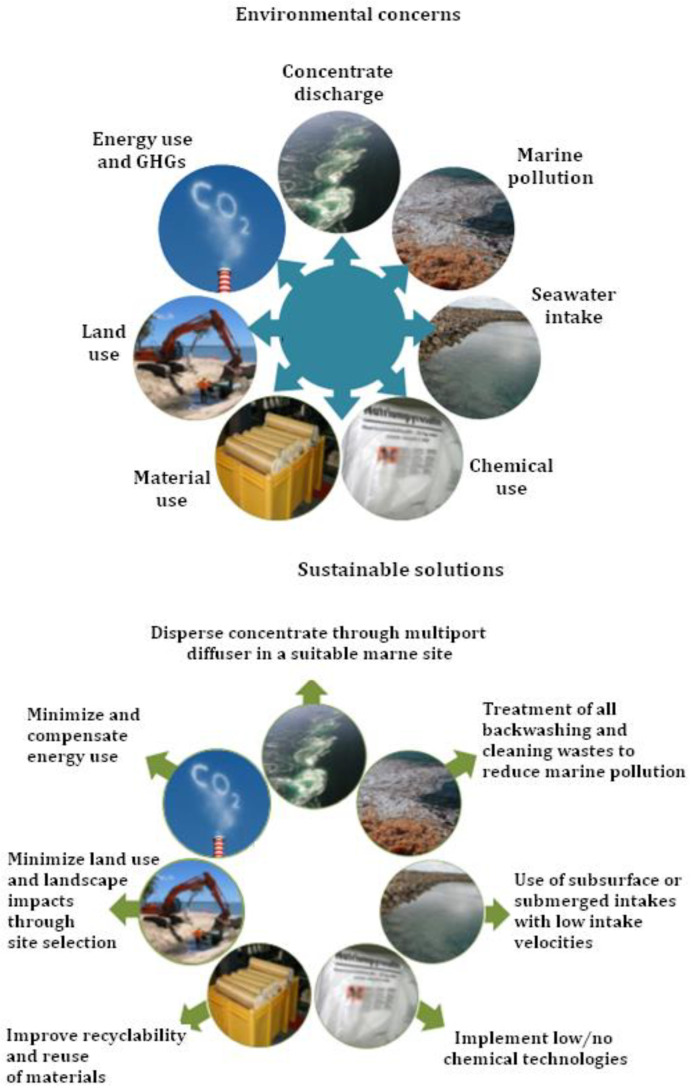
Environmental concerns and sustainable solutions for the desalination plant to minimize the environmental impact [28].

The summary of the environmental challenges and possible sustainable solutions is illustrated in Figure 9. 

To evaluate the potential environmental impact of the water desalination plants, a life cycle assessment (LCA) tool can be a useful tool for application across the whole life cycle [30,31]. The LCA takes into account all the phases of a product’s life cycle, starting from the acquisition of raw materials to the end-of-life phase (collection/sorting, reuse, recycling, waste disposal) [30]. The LCA technique has four phases: goal and scope definition, inventory analysis, impact assessment, and interpretation [32]. There is various software available that can be applied to the LCA, such as EIME V5, Cycle IT System V1.1, e-LICCO, Open LCA 1.2, GaBi, SimaPro Analyst 7.3.3, Umberto 5.6, and others [30].

### 5.3. Membrane Fouling and Scaling 

Membrane fouling is still the main “Achilles heel” for the cost-effective application of reverse osmosis [21]. The types of fouling are categorized into (i) particulate/colloidal fouling, (ii) inorganic fouling (scaling), and (iii) organic and biofouling. To prevent the occurrence of membrane fouling, SWRO plants are always equipped with pre-treatment systems (e.g., media filters with coagulation), microfiltration/ultrafiltration, etc. Moreover, the particulate and colloidal fouling are mostly controlled by this improvement in the pre-treatment phase; however, the occurrence of organic and biofouling is still a major issue in SWRO membranes, the consequences of which are:

- increase in head loss across the feed spacer of spiral wound elements
- higher energy consumption to maintain the constant flux operation
- higher chemical cleaning frequency
- increased frequency of membrane replacement due to irreversible membrane fouling
- decreased rate of water production due to longer downtime during chemical cleaning and membrane replacement
- increased salt passage, thus deteriorating the permeate quality

## 6. Concluding Remarks

By 2050, about 44 countries (2 billion people) will be strongly affected by water scarcity, and more than 95%, or 1.9 billion, of these people will live in developing countries. These countries are Uganda, Burundi, Nigeria, Somalia, Malawi, Eritrea, Ethiopia, Haiti, Tanzania, Niger, Zimbabwe, Afghanistan, Sudan, and Pakistan.

Currently, the majority of these 13 water-scarce countries have not yet installed desalination to meet their freshwater demands. By 2050, the projected desalination capacity of 57 Mm^3^/d will be required in these countries in order for them to meet the standard of their current water demand and to compensate for the withdrawal of renewable resources. This projection assumes that no withdrawal of renewable water resources will occur in these countries, and that the growing urban population water demand will be fully supplied by desalination. Furthermore, the current average water withdrawal for municipal purposes was assumed to be constant throughout the projection period. 

The experiences of some countries (e.g., India, China, and South Africa) in using desalination water for industrial users may be adopted in other nations to solve the issue of water scarcity by 2050.

The current global trend shows that desalination technology is finding new outlets as an alternative source for supplying water to meet growing water demand in most of the water-scarce countries. However, there have been barriers to the widespread adoption of this technology mainly due to its cost, energy requirements, lack of expertise, and its large carbon footprint.

## Figures and Tables

**Table 1:** Currently installed desalination capacity (sea and brackish water) in the chosen water-scarce countries and its share of municipal and domestic supply [3].

Country	Region	Desalination Capacity, Q_2020_			
		Seawater		Brackish Water	
		Capacity, Q_SW_, [m^3^/Day × 1000]	Municipal Water, Y_SW_	Capacity, Q_BW_, [m^3^/Day × 1000]	Municipal Water, Y_BW_
Afghanistan	Central Asia	0.00	0.00	2.5	0.85
Burundi	East Africa	0.00	0.00	0.00	0.00
Eritrea	Northeast Africa	1.00	0.00	0.15	0.00
Ethiopia	Northeast Africa	1.72	0.42	1.82	0.01
Malawi	Southeast Africa	0.00	0.00	0.00	0.00
Niger	West Africa	0.00	0.00	0.40	0.00
Nigeria	West Africa	13.28	0.45	115.61	0.01
Pakistan	South Asia	85.59	0.37	104.90	0.04
Somalia	East Africa	0.12	0.00	0.00	0.00
Sudan	North Africa	43.25	0.82	0.48	0.00
Tanzania	East Africa	0.60	1.00	6.08	0.00
Uganda	East Africa	0.00	0.00	0.00	0.00
Zimbabwe	Southern Africa	0.00	0.00	0.00	0.00

**Table 2:** Water withdrawal in each water-scarce country [15].

Countries	N_2020_ [Millions]	Urban Population [Millions]	Water Withdrawal, m^3^/Capita/Day			
			Agriculture	Municipal	Industries	Total
Afghanistan	38.93	1.32	9.13	0.08	0.08	9.30
Burundi	11.89	1.54	0.05	0.08	0.00	0.06
Eritrea	3.21	0.67	0.47	0.13	0.00	0.50
Ethiopia	114.96	18.39	0.23	0.12	0.00	0.25
Malawi	19.13	3.63	0.17	0.11	0.01	0.19
Niger	24.21	4.12	0.17	0.12	0.00	0.20
Nigeria	206.14	101.01	0.07	0.14	0.03	0.17
Pakistan	220.89	79.52	2.14	0.33	0.02	2.27
Somalia	15.89	5.88	0.57	0.01	0.00	0.57
Sudan	43.85	17.10	1.62	0.15	0.00	1.68
Tanzania	59.73	15.53	0.21	0.09	0.00	0.24
Uganda	45.74	5.95	0.02	0.15	0.00	0.04
Zimbabwe	14.86	5.65	0.56	0.31	0.02	0.70
Average			1.19	0.140	0.01	1.24

**Table 3:** The current installed and projected desalination capacity for sea and brackish water desalination plants, m^3^/d, in the selected water-scarce countries.

Country	N_2020_ [Millions]	N_2050_ [Millions]	Urban Population	Q_SW_	Y_SW_	Q_BW_	Y_BW_	Q_2020_ [m^3^/cap/d] × 1000	WW_AVG_ [m^3^/cap/d]	∆Q_2050_ [Mm^3^/d]
			[% Share]	[m^3^/d × 1000]		[m^3^/d × 1000]				
Afghanistan	38.93	69.5	22%	0.00	0.00	2.50	0.85	0.25	0.140	2.13
Burundi	11.89	19.5	11%	0.00	0.00	0.00	0.00	0.00	0.140	0.30
Eritrea	3.21	10.5	21%	1.00	0.00	0.15	0.00	0.00	0.140	0.31
Ethiopia	114.96	171	16%	1.72	0.42	1.82	0.01	0.04	0.140	3.82
Malawi	19.13	25.9	19%	0.00	0.00	0.00	0.00	0.00	0.140	0.69
Niger	24.21	53	17%	0.00	0.00	0.40	0.00	0.00	0.140	1.26
Nigeria	206.14	258.5	49%	13.28	0.45	115.61	0.01	0.07	0.140	17.69
Pakistan	220.89	348.7	36%	85.59	0.37	104.90	0.04	0.45	0.140	17.50
Somalia	15.89	39.7	37%	0.12	0.00	0.00	0.00	0.00	0.140	2.05
Sudan	43.85	60.1	39%	43.25	0.82	0.48	0.00	2.08	0.140	3.24
Tanzania	59.73	69.1	26%	0.60	1.00	6.08	0.00	0.04	0.140	2.51
Uganda	45.74	103.2	13%	0.00	0.00	0.00	0.00	0.00	0.140	1.87
Zimbabwe	14.86	23.5	38%	0.00	0.00	0.00	0.00	0.00	0.140	1.25
Total	819.45	1252.20	-	145.56	-	231.93	-	2.92	-	57.1
Average	63.03	96.32	-	11.20	-	17.84	-	0.22	-	4.20

**Table 4:** Comparison of water costs for conventional and desalination water supply options (Voutchkov, 2011, 2014; Plappally, 2012) as cited by [26].

Range	Energy Requirements (kWh/m^3^)		Water Production Costs ($/m^3^)	
	Conventional Water Supplies	Seawater Reverse Osmosis (SWRO)	Conventional Water Supplies	Seawater Reverse Osmosis (SWRO)
Low	0.1–0.5	2.5–2.8	0.25–0.75	0.5–0.8
Medium	1.0–2.5	3.0–3.5	0.75–2.50	1.0–1.5
High	2.5–4.5	4.0–4.5	2.50–5.00	2.0–4.0
